# Diverse Effects of Various Toll-Like Receptor 2 Ligands on Neuronal Activity and Cell Death

**DOI:** 10.1007/s10571-025-01632-3

**Published:** 2025-11-26

**Authors:** Futa Sato, Satoshi Hachimura

**Affiliations:** 1 Research Center for Food Safety, Graduate School of Agricultural and Life Sciences, The University of Tokyo, 1-1-1 Yayoi, Bunkyo-ku, Tokyo, 113-8657 Japan; 2 Department of CNS Research, Otsuka Pharmaceutical Co, Ltd, 463-10 Kagasuno, Kawauchi-cho, Tokushima-shi, Tokushima, 771-0192 Japan

**Keywords:** TLR2, Neuron, Cell death, Calcium oscillation

## Abstract

The role of Toll-like receptor 2 (TLR2) in the central nervous system (CNS) is critical in several conditions including neurological disorders such as pain, and neurodegenerative disorders such as Parkinson’s disease. Therefore, understanding TLR2 function in the CNS is of considerable importance. In this study, we investigated neuronal responses to individual TLR2 ligands. The expression levels of cytokines increased in the culture in the presence of TLR2 ligands. Additionally, increased lactate dehydrogenase (LDH) was noted during lipoteichoic acid (LTA) stimulation. During LTA stimulation, a decrease in the peak amplitude of Ca^2 +^ oscillations was observed. MnTBAP, which is a reactive oxygen species (ROS) blocker, inhibited the LTA-induced cell death but had no effect on the peak amplitude of the Ca^2 +^ spike. Conversely, Pam3CSK4 (P3C) stimulation increased the number of Ca^2 +^ peaks, which was inhibited by a tumor necrosis factor alpha (TNFα) signaling inhibitor. Our study revealed that several TLR2 ligands, each with different specificities, elicited diverse responses in primary cortical cells. In conclusion, TLR1-TLR2 and TLR2-TLR6 signaling reduces the peak amplitude and induces cell death, and TLR1-TLR2 signaling enhances Ca^2 +^ dynamics via a TNFα pathway.

**Graphical Abstract:**

**Figure:**
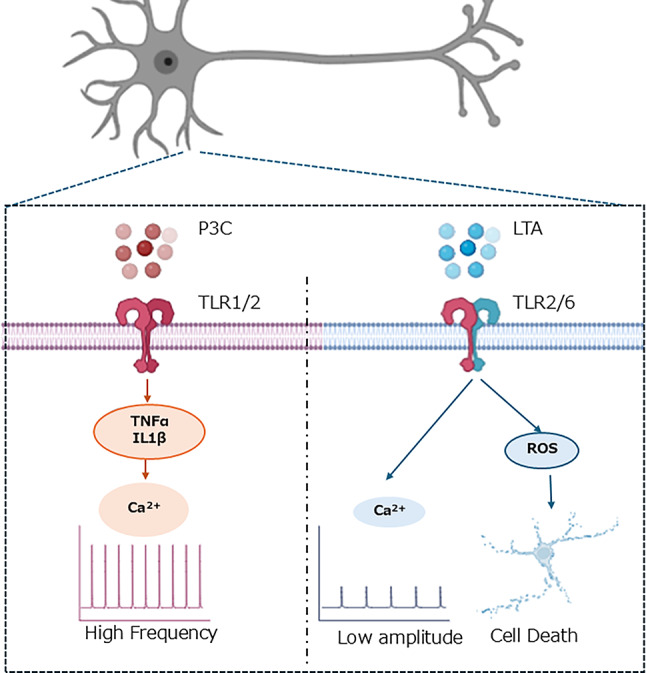
P3C stimulation increased the number of Ca^2+^ peaks, which was inhibited by a TNFα signaling inhibitor. LTA stimulation decreased the amplitude of Ca^2+^ peaks, and induced cell death via ROS signaling.

**Supplementary Information:**

The online version contains supplementary material available at 10.1007/s10571-025-01632-3.

## Introduction

Many pathogens possess common pathogen/damage-associated molecular patterns (PAMPs/DAMPs), and immune cells recognize these PAMPs/DAMPs through pattern recognition receptors (PRRs) (Donnelly et al. 2020). TLRs, which are a type of PRR, play pivotal roles in the immune response against bacterial infections (Fitzgerald and Kagan 2020; Li and Wu 2021). The involvement of TLR signaling has been reported in relation to infections by viruses and bacteria in the CNS (Aravalli et al. 2007). It is well known that TLR2 recognizes a variety of bacterial lipopeptides, such as LTA (Schwandner et al. 1999), synthetic lipoprotein structure P3C (Funderburg et al. 2011), and bacterial lipopolysaccharide (LPS) (Kirschning et al. 1998). Previous studies have reported that a neuronal cell line upregulate cytokine gene expression via TLR2 (Dzamko et al. 2017) and that both neuronal cells and microglia express TLR2 in mouse brain tissue (Kim et al. 2013). It has also been reported that amyloid β enhances interleukin (IL)-8 production via TLR2-TLR1 and TLR2-TLR6 heterodimers in HEK293 cell lines engineered to overexpress TLR1, TLR2, and TLR6 (Liu et al. 2012). TLR2 ligands commonly used in in vitro assays have different specificity for TLR dimers and accessory molecules and have different specificity within the TLR family. P3C can stimulate the TLR1-TLR2 dimer (Cheng et al. 2015). LPS can bind the TLR2-TLR4 heterodimer (Francisco et al. 2021). Zymosan A (ZA) can stimulate the TLR2-TLR6 heterodimer (Ozinsky et al. 2000). LTA can stimulate TLR1-TLR2 and TLR2-TLR6, but not TLR4 (Triantafilou et al. 2006). The TLR2-TLR6 heterodimer uses CD14 to respond to ZA and both CD14 and CD36 to respond to LTA (Lee et al. 2012). However, there are few studies that investigate the role of TLR2 in Ca^2 +^ dynamics and cell death.

Regarding TLR2 and neurons in vitro, the following findings have been reported. There are reports that TLR2 is expressed in human and mouse cortical neurons (Tang et al. 2007; Zhou et al. 2009). A previous study has shown that TLR2 signaling is required for the production of proinflammatory cytokines in a neuronal cell line (Chung et al. 2022). Furthermore, it is known that TLR2 induces neuronal cell death through the induction of cytokines in astrocytes (Goshi et al. 2022; Kinsner et al. 2005). Previous studies have shown that mature neurons are less sensitive to stimulation that induces neuronal death than immature neurons (Barreda-Manso et al. 2023). Organotypic hippocampal slice cultures have been used to study the attenuation of excitatory postsynaptic potentials by stimulation of TLR2 in neurons (Costello et al. 2011). Ca^2 +^ influx by TLR2 ligands has been reported in peripheral nerves (T.-T. Wang et al. 2020). Inflammatory cytokines have been reported to induce changes in neural network activity measured by waveform analysis (Clarkson et al. 2017). It has been reported that amyloid beta induced cell death using a mouse NG108-15 neural cell line (Lin et al. 2013). It would be valuable to investigate the detailed effects of TLR2 on nervous system cells using primary cell cultures.

Neuroinflammation is thought to be an important factor in some CNS disease animal models, and the involvement of TLR2 expression in brain tissue has been reported (Squillace and Salvemini 2022). It has been reported that avoidance behavior in a social defeat stress model is not observed in CX3CR1 specific TLR2-TLR4 double knockout mice (Nie et al. 2018). The impairment of memory-related functions induced by TLR2 stimulation has been demonstrated through experiments using neural progenitor cells as well as mouse behavioral studies (Madar et al. 2015). The expression or knockdown of TLR2 has been reported to contribute to the accumulation of α-synuclein and cell death in the neuronal cell line SH-SY5Y (Kim et al. 2015). In a neuropathic pain model, it has been reported that less pain behavior is induced in TLR2 knockout animals (Shi et al. 2011; Zhang et al. 2025). It is believed that TLR2 significantly affects the CNS; therefore, to address the role of TLR2 under a broad range of disease-relevant conditions, including infection and exposure to aggregated proteins, we investigated the effects of TLR2 stimulation in primary neuronal cultures. This in vitro approach allows us to isolate neuron-intrinsic responses and mechanistically assess how TLR2 signaling modulates neuronal excitability and vulnerability to cell death.

It is well established that fluctuations in calcium levels are correlated with electrical neural activity (Smetters et al. 1999). The development of Ca^2 +^ indicators and detectors has made it possible to analyze the phenotypes of Ca^2 +^ dynamics in neurons (Sakaguchi et al. 2023). In this article, we report the function of TLR2 in Ca^2 +^ dynamics. We investigated TLR2-mediated neuronal cell death and the changes in neuronal activity mediated by TLR2 and proinflammatory cytokines, as demonstrated in previous studies using animal models and cell lines. For this purpose, we utilized primary cultured neuronal cells to further examine these mechanisms. We were able to demonstrate that TLR2 affects Ca^2 +^ dynamics and cell death. These findings provide insights into the function of inflammation in several models of mental and neurodegenerative diseases.

## Materials and Methods

### Animals

All rat experiments in this study were approved by the Animal Care and Use Committee for Otsuka Pharmaceutical Co., Ltd. (Approval number: 21–0156, 22–0147, 23–0106)

All animal care and experimental procedures were carried out in accordance with the “Guidelines for Animal Care and Use” issued by Otsuka Pharmaceutical Co., Ltd.

### Cell Cultures

Rat primary cortical neuronal cultures were obtained from Crl: CD (SD) rat embryos at embryonic day 18. The cortical tissues were dissected using the Papain Dissociation System (Worthington Biochemical Corp). Cortical tissues were collected in cold Hanks’ balanced salts solution (HBSS) and dissociated in 0.25% papain/DNase I in phosphate-buffered saline (PBS) for 30 min in an incubator. These samples were added to 10 mL of neurobasal medium and pipetted to dissociate the cells. The supernatant was collected, filtered using a cell strainer (#352350, Falcon), and centrifuged (1,000 rpm, 5 min). The pellet was suspended in ovomucoid protease inhibitor/HBSS, centrifuged (800 rpm, 5 min), and resuspended in the medium for harvesting.

These cells were cultured on poly-D-lysine pre-coated 96-well plates (Merck) at a density of 50,000 cells/well. Cells were cultured at 37°C in neurobasal medium containing supplements (100 µg/mL penicillin and streptomycin (Thermo), 5% B27 Supplement (Thermo), and 10% GlutaMax (Thermo)). Half of the medium was replaced once every 2–4 days.

These cells were treated with distilled water (DW) as control, LTA (pan-TLR2 agonist), P3C (TLR1-TLR2 agonist), LPS (TLR4(-TLR2) agonist, InvivoGen), ZA (pan-TLR2 agonist, FUJIFILM WAKO PURE CHEMICAL). Simultaneously, these cells were cultured in the presence of TLR2 ligands and MnTBAP (Abcam), a ROS blocker, R-7050 (MedChem Express), a TNFα signaling inhibitor, or tofacitinib, a Janus kinase (JAK) inhibitor involved in IL-6 signaling. Figure S1 shows an overview of the experimental workflow.

### Immunocytochemistry

Cultured cortical cells were fixed with 4% paraformaldehyde (PFA, FUJIFILM WAKO PURE CHEMICAL) for 20 min at room temperature followed by washing with PBS. After removing the PFA, fixed cells were treated with the blocking and permeabilization buffer containing 0.1% Triton X-100 (SIGMA) for 30 min at room temperature. After aspiration of the blocking and permeabilization buffer, fixed cells were treated with a primary antibody overnight at 4°C. Each well was washed with 0.1% bovine serum albumin (BSA) in PBS and was treated with a secondary antibody for 2 h at room temperature. Each well was washed by 0.1% BSA in PBS again. Fixed cells were treated with 0.1% BSA in PBS containing Hoechst 33342. Anti-class III beta-tubulin (TUBB3) antibody (R&D systems, MAB1195), a marker for neurons (Ballas et al. 2005), anti-TLR2 antibody (Thermo, MA532787), anti-glial fibrillary acidic protein (GFAP) antibody (abcam, ab68428), a marker for astrocytes (Shixing et al. 2023), anti-Iba1 antibody (CST, 17198T), a marker for microglia (Ni et al. 2022), and anti-Olig2 antibody (abcam, ab109186), a marker for oligodendrocytes (Xu et al. 2023), were used as primary antibodies (Wyczanska et al. 2023). Rabbit Alexa Fluor 568 (Thermo, A11011) and Mouse Alexa Fluor 488 (Thermo, A21202) were used as secondary antibodies. Images of stained cells were acquired by CellVoyager CV8000 (Yokogawa Electric Corporation) equipped with a 20×objective lens (Olympus, Tokyo, Japan). Three channels were employed to visualize the various fluorescent stains: DNA (excitation: 405 nm; emission: 445/45 nm), TUBB3 (excitation: 488 nm; emission: 525/50 nm), TLR2 and other non-neuronal markers (excitation: 561 nm; emission: 676/29 nm). Four fields of view were captured per well. This experiment was performed nonblinded because fluorescent images were automatically acquired using CellVoyager CV8000.

### Lactate Dehydrogenase Assay

The amount of LDH released in the supernatants was assessed using the LDH Cytotoxicity Kit (Nacalai) in accordance with the instructions in the manufacturer’s manuals (Lobner 2000). We harvested 50 µL/well of culture supernatant and transferred this to an assay plate. The measurement samples were diluted twofold by adding 50 µL/well of fresh medium. Next, 100 µL/well of substrate solution was added to the diluted samples. The plates were then incubated at room temperature for 20 min, protected from light. Following incubation, 50 µL/well of stop solution was added to all wells. Absorbance was measured at 490 nm using a plate reader, and the results are presented graphically.

### Ca^2 +^ Assay

Cal-520 AM (AAT Bioquest) was prepared at 2 µM in HBSS and 10 mM HEPES buffer (Thermo) containing 0.01% Pluronic F-127 (BIOTIUM). The cortical cells were incubated with dye-loading solution for 60 min in a 5% CO_2_ incubator at 37°C. The calcium oscillation assays were performed on FDSSµCELL (HAMAMATSU PHOTONICS K.K.). The results of the assays were analyzed using SpotFire and WaveFinder (PerkinElmer) (Sakaguchi et al. 2023). A point is considered the start of a peak if its value is higher than the values of the preceding ten points. The subsequent local maximum is then designated as the peak top.

### qPCR Analysis

The TaqMan™ Fast Advanced Cells-to-CT™ Kit (Thermo) was used for RNA isolation from cultured cells, and cDNA synthesis. Quantitative PCR was performed with TaqMan primers (Thermo, Assay ID. Rn00580432_m1 < *Il1b*>, Assay ID. Rn01410330_m1 < *Il6*>, Assay ID. Rn00572010_m1 < *Tgfb1*>, Assay ID. Rn01525859 < *Tnf*>, Assay ID. Rn99999916_s1 < *Gapdh*>) and the TaqMan™ Fast Advanced Cells-to-CT™ Kit in accordance with the manufacturer’s instructions (Rodriguez-Rodriguez et al., 2024). *Gapdh* was used for normalization.

### Statistical Analysis

Statistical analysis of each experimental feature was performed with SAS Software version 9.4 for Windows (SAS institute) and Microsoft Excel for Microsoft 365 MSO. Figures were created using GraphPad Prism 10.3.0 for Windows (GraphPad Software). No priori sample size calculation was performed. The results represent the evaluation of 2 or 4 wells (2 or 4 biological replicates within 1 technical replicate) in a single experiment. The experiment was repeated independently on two separate occasions to ensure reproducibility. Statistical analysis was performed using Dunnett’s test to compare each treatment group with the control group, which is appropriate for multiple comparisons against a single control. Tukey’s test was used for pairwise comparisons among all experimental groups, including both the control and TLR ligand-treated conditions, to evaluate the relative effects of the compounds.

## Results

### The Primary Rat Cortical Cells Express TLR2

**Fig. 1:**
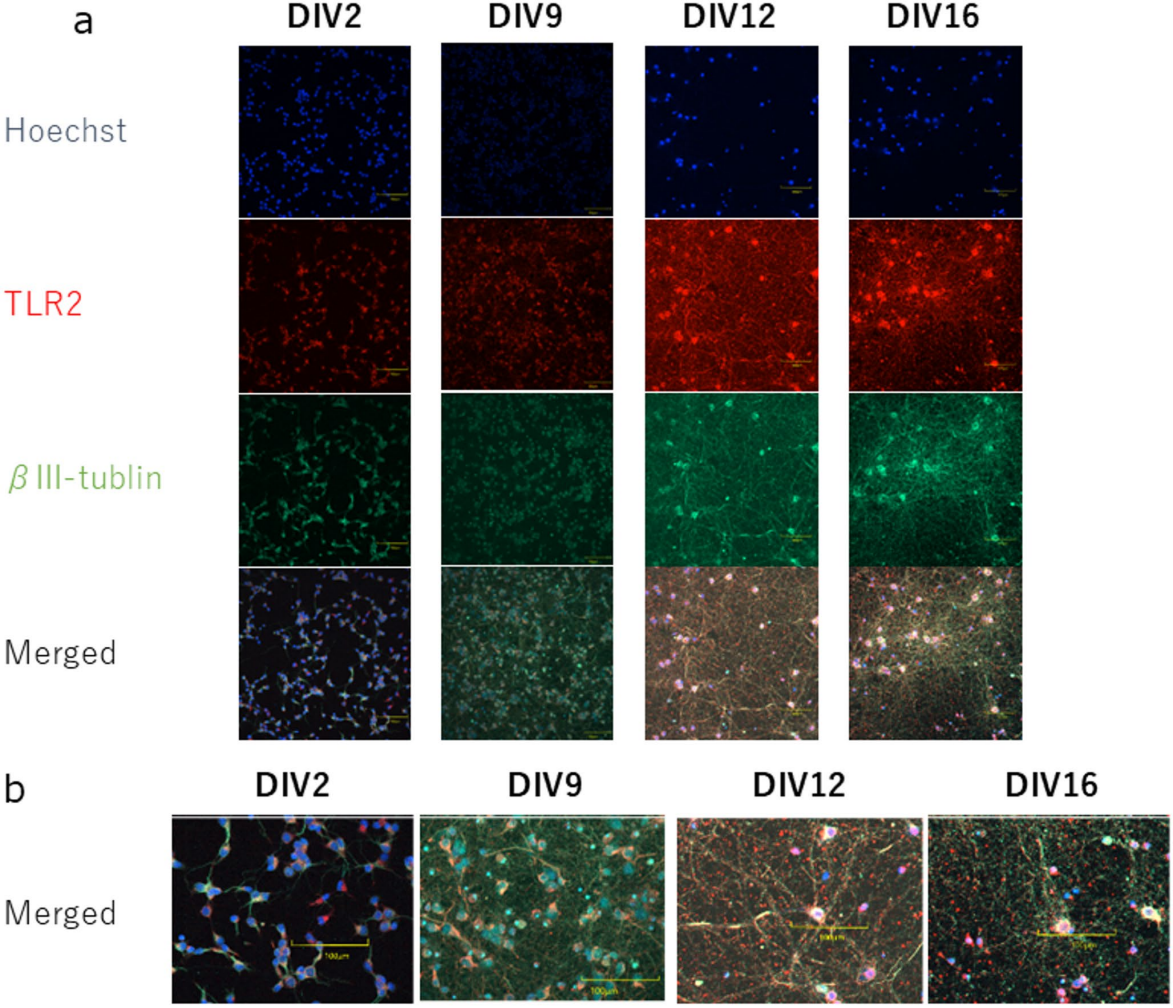
Expression of TLR2 in the cortical-derived neurons and other cells. Primary rat cortical cells were cultured for 2–16 days. (**a**) Representative images of primary cortical cells at DIV2, DIV9, DIV12, and DIV16. Representative images showing the signal from Hoechst 33342-stained nuclei in blue, beta III tubulin immunoreactivity in green, and TLR2 immunoreactivity in red in the cultured cortical cells, and merged image (bottom panel). (4 biological replicates in 1 technical replicate from 2 independent experiments) (**b**) Magnified images from (**a**) showing TLR2 in neurons. Scale bar: 100 μm. Cells were analyzed in 4 fields of view. Two independent experiments were performed

Cortical cells containing mouse neurons and microglia are known to express TLR2 (Kim et al. 2015). To confirm that the cortical cells express TLR2 in this rat culture system, we investigated TLR2 immunocytochemistry. According to Kinsner et al., cell death induced by LTA stimulation for more than three days has been reported. Additionally, Pacico et al. reported calcium oscillations occurring at days in vitro (DIV)12 and beyond. Based on these findings, we decided to investigate the influence of TLR2 across the time frame from DIV2 to DIV16. We fixed the cultured cells with 4% PFA at DIV2, DIV9, DIV12, and DIV16. We next performed immunostaining with an anti-TLR2 antibody, an anti-beta III tubulin, as a neuron marker, and Hoechst 33342 as a nucleus marker. We found that cortical cells expressed TLR2 between DIV2 and 16 (Fig. 1). These observations also indicated that both cortical neurons and non-neuronal cells express TLR2 in our primary rat cortical cell culture system (Fig. 1b). These observations are consistent with a previous report using a mouse culture system. The absence of microglia and oligodendrocytes was confirmed via Iba1 and Olig2 staining, respectively. GFAP staining revealed a sparse population of astrocytes, with few GFAP-positive cells per microscopic field, suggesting minimal but non-negligible glial presence (Fig. S2).

### LTA Induces Cell Death

**Fig. 2:**
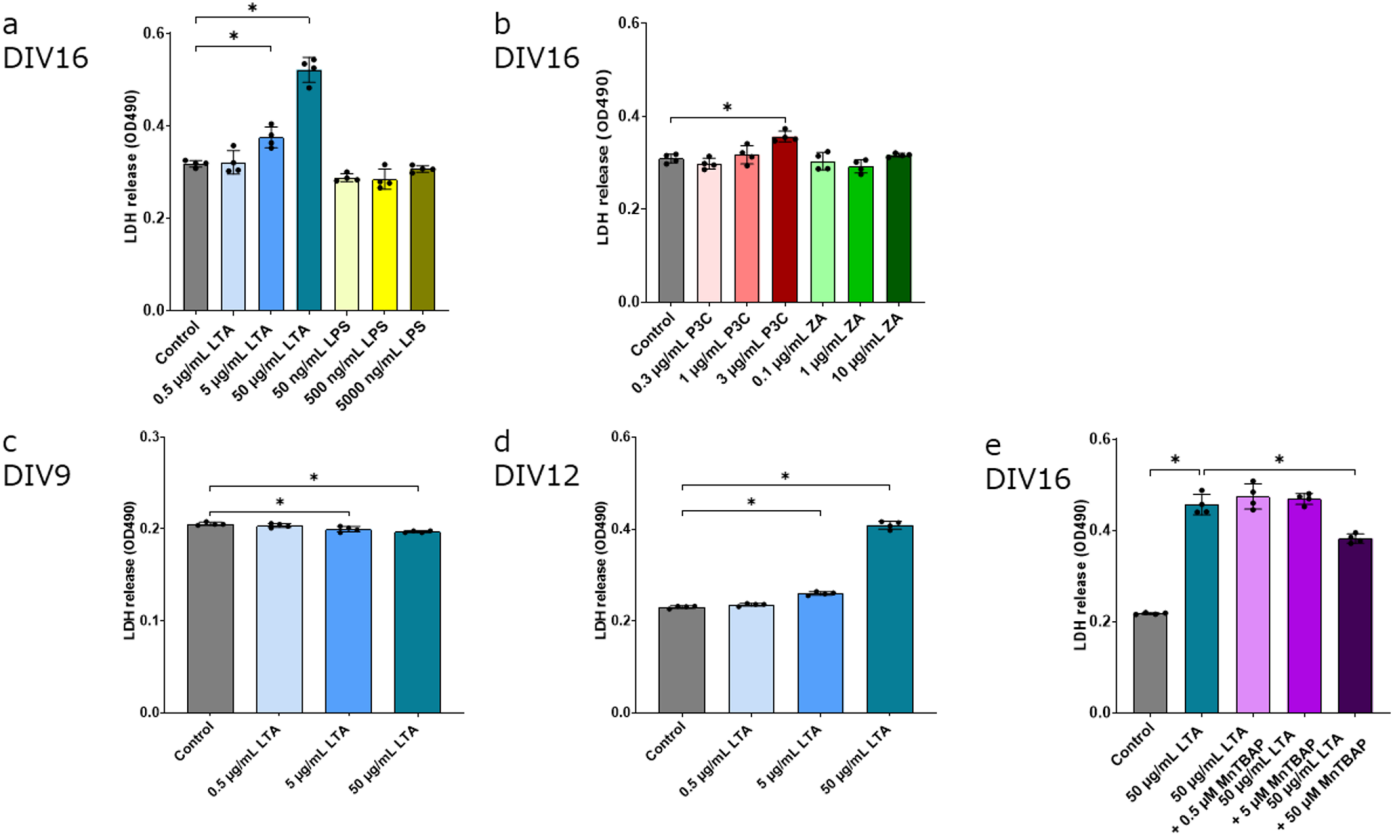
The effect of TLR2 ligands on LDH release in neuronal cultures via ROS. (**a**–**d**) Release of LDH was measured photometrically as a surrogate for cell death after treatment with TLR2 ligands. Control cultures were treated with control. (4 biological replicates in 1 technical replicate from 1 of 2 independent experiments) (**a**) The cells were cultured for DIV2–16 in the presence of LTA and LPS. (**b**) The cells were cultured for DIV2–16 in the presence of P3C and ZA. (**c**) The cells were cultured for DIV2-9 in the presence of LTA. (**d**) The cells were cultured for DIV2-12 in the presence of LTA. (**e**) The cells were cultured between DIV2 and DIV16 in vitro in the presence of 50 µg/mL LTA and MnTBAP. The data are presented as the mean ± SD. The results represent the evaluation of four wells (4 biological replicates in 1 technical replicate) in a single experiment. To ensure reproducibility, the experiment was independently repeated twice. In these LDH assays, two independent experiments were performed. *P < 0.05 vs. control group as determined by Dunnett’s test (**a**–**d**). *P < 0.05 vs. LTA group as determined by Tukey’s test (**e**)

LTA, one of the TLR2 ligands, is known to induce cortical cell death through ROS stress (Kinsner et al. 2005). To further understand the relationship between TLR2 and cell death, we investigated whether several TLR2 ligands could trigger cell death. We treated the plated neuronal cells with TLR2 ligands at DIV2. We assessed cell death at DIV16 using the LDH assay. Figure 2 shows that LTA induced cell death but LPS (Fig. 2a), ZA (Fig. 2b) did not. P3C slightly increased LDH (Fig. 2b). These results suggested that TLR2-induced cell death depends on the ligand specificity and efficacy. Based on the study by Pacico et al., we focused on DIV9, DIV12, and DIV16, which correspond to the periods shortly before and after the onset of calcium oscillations, and detected cell death during these time points. When we treated the cortical cells with LTA, the amount of LDH slightly decreased at DIV9 (Fig. 2c), and the amount of LDH increased at DIV12 (Fig. 2d). To investigate the time-dependent effects of LTA stimulation, we additionally explored multiple time points in an exploratory manner. LTA can induce cell death when LTA treatment started at DIV9, but not at DIV12 (Fig. S3). These results reveal that LTA promotes cell death through mechanisms that are dependent on both concentration and time.

A previous study showed that ROS blockers can suppress LTA-induced cell death (Kinsner et al. 2005). It has been reported that TNFα induced neuronal cell death (Olianas et al. 2019). We investigated the relationship between cell death and TLR2 signaling. We treated TLR2 ligands and MnTBAP with plated neuronal cells at DIV2. We assessed the cell death at DIV16 using the LDH assay. In Fig. 2e, the Tukey test indicated that the 50 µg/mL LTA + 50 µM MnTBAP was significantly lower than the 50 µg/mL LTA (*p* < 0.001).

### TLR2 Ligands Regulate Ca^2 +^ Dynamics

**Fig. 3:**
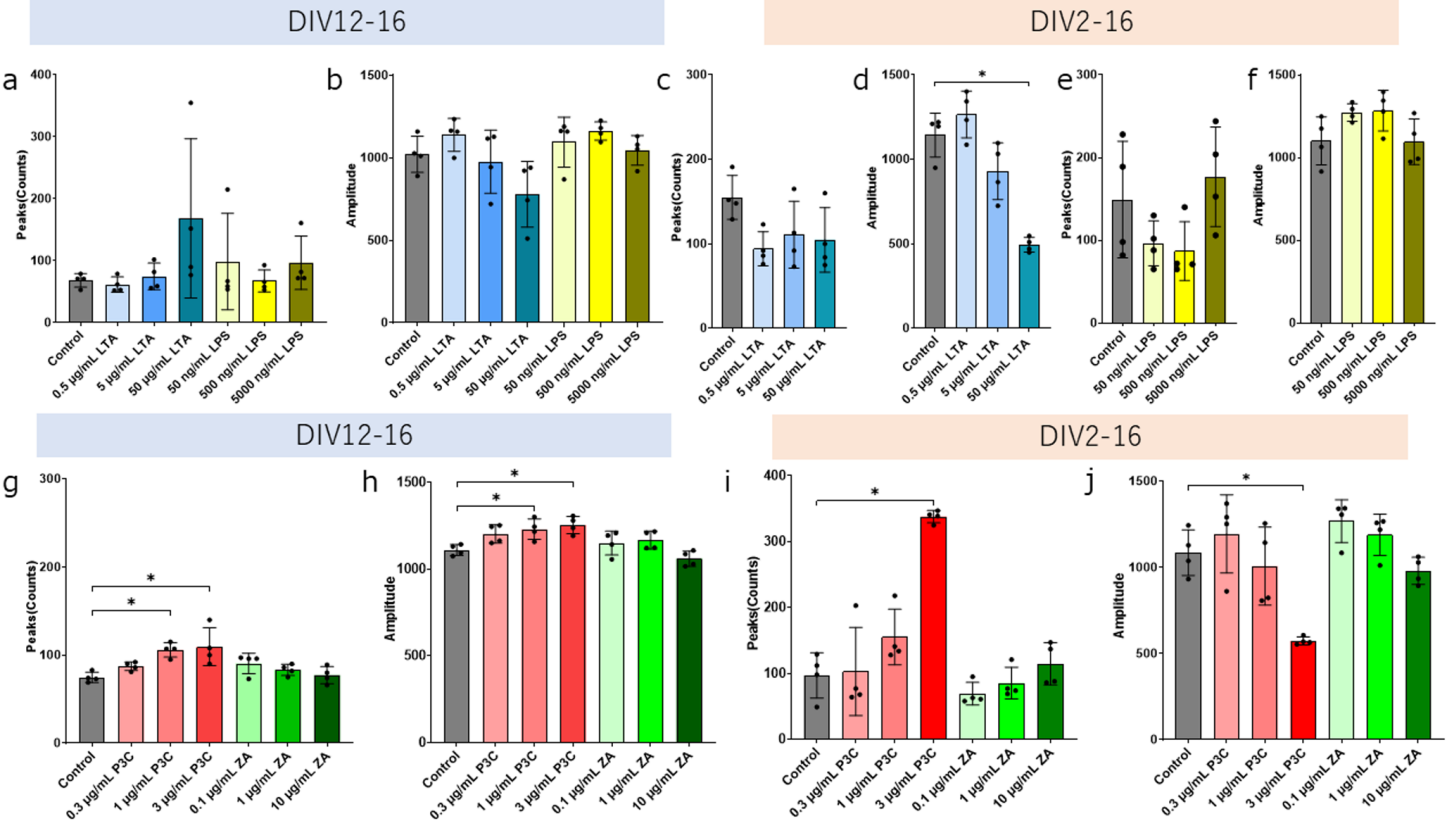
TLR2 ligands have different effects on the Ca^2 +^ oscillation. The cells were treated with LTA (**a**¬**d**) or LPS (**a**, **b**, **e**, **f**), P3C or ZA (**g**–**j**) at DIV12 (**a**, **b**, **g**, **h**) or DIV2 (**c**–**f**, **i**, **j**) (The results represent the evaluation of four wells in a single trial. To ensure reproducibility, the experiment was independently repeated twice.). Control cultures were treated with 0.1% DW. Cal-520 was loaded into the cells at DIV16 and detected by FDSSµCELL. Calcium spikes were analyzed by WaveFinder (PerkinElmer). Each graph shows the peaks of Ca^2 +^ spikes or the amplitude of Ca^2 +^ spikes. The data are presented as the mean ± SD. In these Ca^2 +^ oscillation assays, two independent experiments were performed. *P < 0.05 vs. control group as determined by Dunnett’s test

A previous study showed that TLR2 ligands affected the concentration of Ca^2 +^ in dorsal root ganglion neurons (T.-T. Wang et al. 2020). Based on our data regarding the expression of TLR2 in cortical cells, we analyzed the Ca^2 +^ oscillation using cortex cells. We conducted a Ca^2 +^ oscillation assay using plated neuronal cells treated with LTA and LPS at DIV12 (Fig. 3a and b) or 2 (Fig. 3c and f, Fig. S4a). Those treated with 50 µg/mL LTA at DIV2 showed significantly smaller amplitude than that of the control group. We conducted a Ca^2 +^ oscillation assay using plated neuronal cells treated with P3C and ZA at DIV12 (Fig. 3g and h) or 2 (Fig. 3i and j, Fig. S4b). Those treated with 1 µg/mL P3C and 3 µg/mL P3C at DIV12 showed significantly more peaks than the control group. Those treated with 3 µg/mL P3C at DIV2 showed significantly more peaks and smaller amplitude than the control group. These results suggest that each TLR2 ligand has different effects on Ca^2 +^ dynamics.

**Fig. 4:**
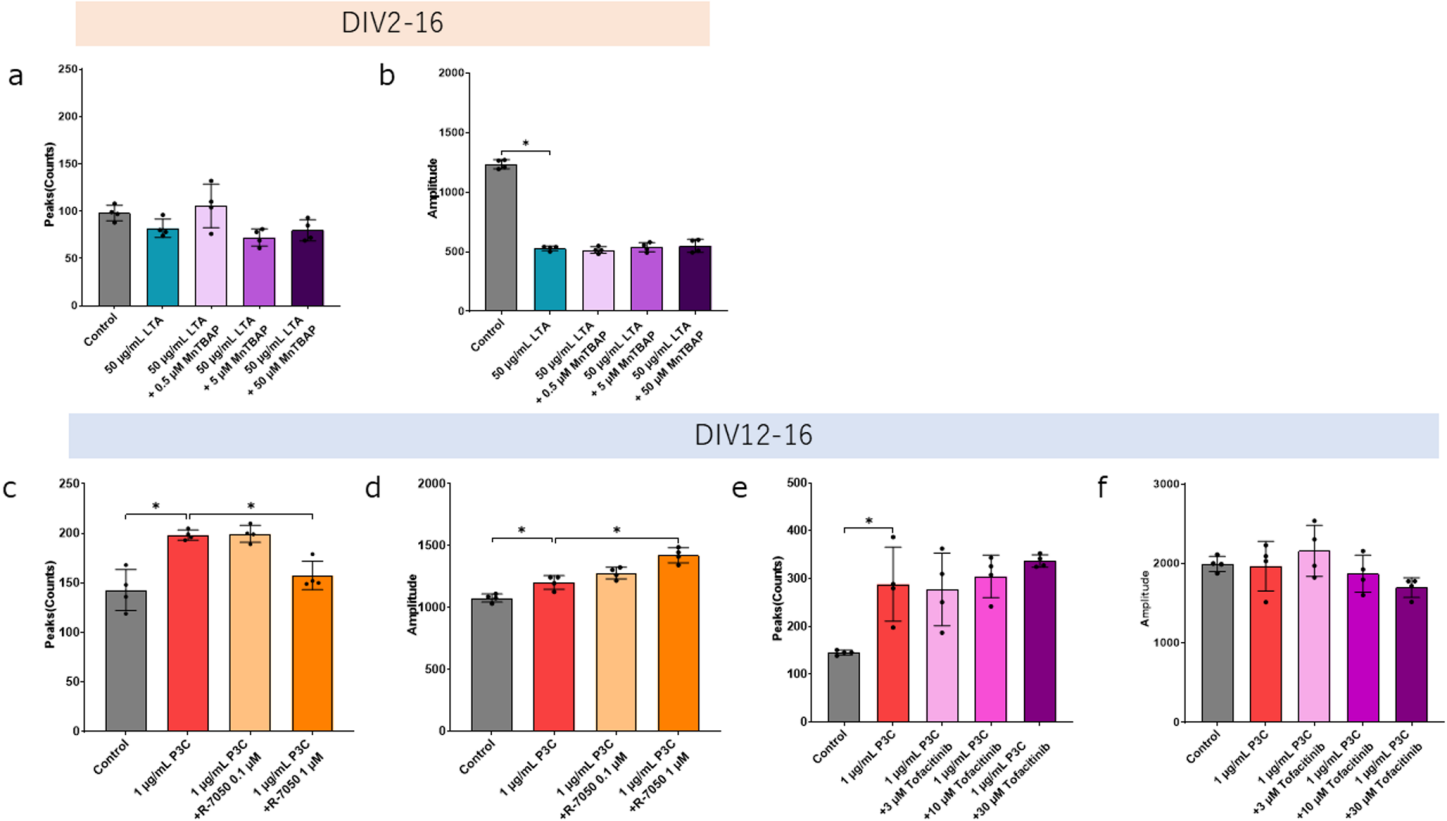
TNFα but not JAK affects peak numbers and ROS does not affect reduction in amplitude. (**a**, **b**) The cells were treated with LTA and MnTBAP. (4 biological replicates in 1 technical replicate from 1 of 2 independent experiments) (**c**, **d**) The cells were treated with P3C and R-7050 at DIV12. (The results represent the evaluation of four wells in a single trial. To ensure reproducibility, the experiment was independently repeated twice.) (**e**, **f**) The cells were treated with P3C and Tofacitinib at DIV12. (4 biological replicates in 1 technical replicate from 1 of 2 independent experiments) Cal-520 was loaded into the cells and detected using FDSS. Calcium spikes were analyzed using WaveFinder. Each graph shows the peaks of Ca^2 +^ spikes or the amplitude of Ca2 + spikes. The data are presented as the mean ± SD. *P < 0.05 vs. LTA or P3C group as determined by Tukey’s test

To confirm whether LTA-induced cell death decreases the amplitude of Ca^2 +^ spikes, we performed a Ca^2 +^ oscillation assay using plated neuronal cells treated with LTA and MnTBAP at DIV2. Figure 4a and b show that MnTBAP did not affect the peaks and amplitude of Ca^2 +^ spikes. Because cytokine signaling has been reported to affect neuronal activity (Tyagi et al. 2024), this led us to hypothesize that TLR2-induced cytokines affect Ca^2 +^ dynamics. We performed a Ca^2 +^ oscillation assay using plated neuronal cells treated with both P3C and a TNFα signaling inhibitor, or both P3C and an IL-6 signaling inhibitor at DIV12. In Fig. 4c, the Tukey test indicated that the 1 µg/mL P3C + 1 µM R-7050 showed significantly less spikes than the 1 µg/mL P3C. Figure 4d, the Tukey test indicated that the peak counts of 1 µg/mL P3C + 1 µM R-7050 were significantly higher than the 1 µg/mL P3C. Figure 4c-f shows that P3C affects Ca^2 +^ dynamics mediated by TNFα signaling, but not by IL-6 signaling.

### Enhancing the Gene Expression of Cytokines in Cortical Cells by Stimulation of TLR2 Ligands

**Fig. 5:**
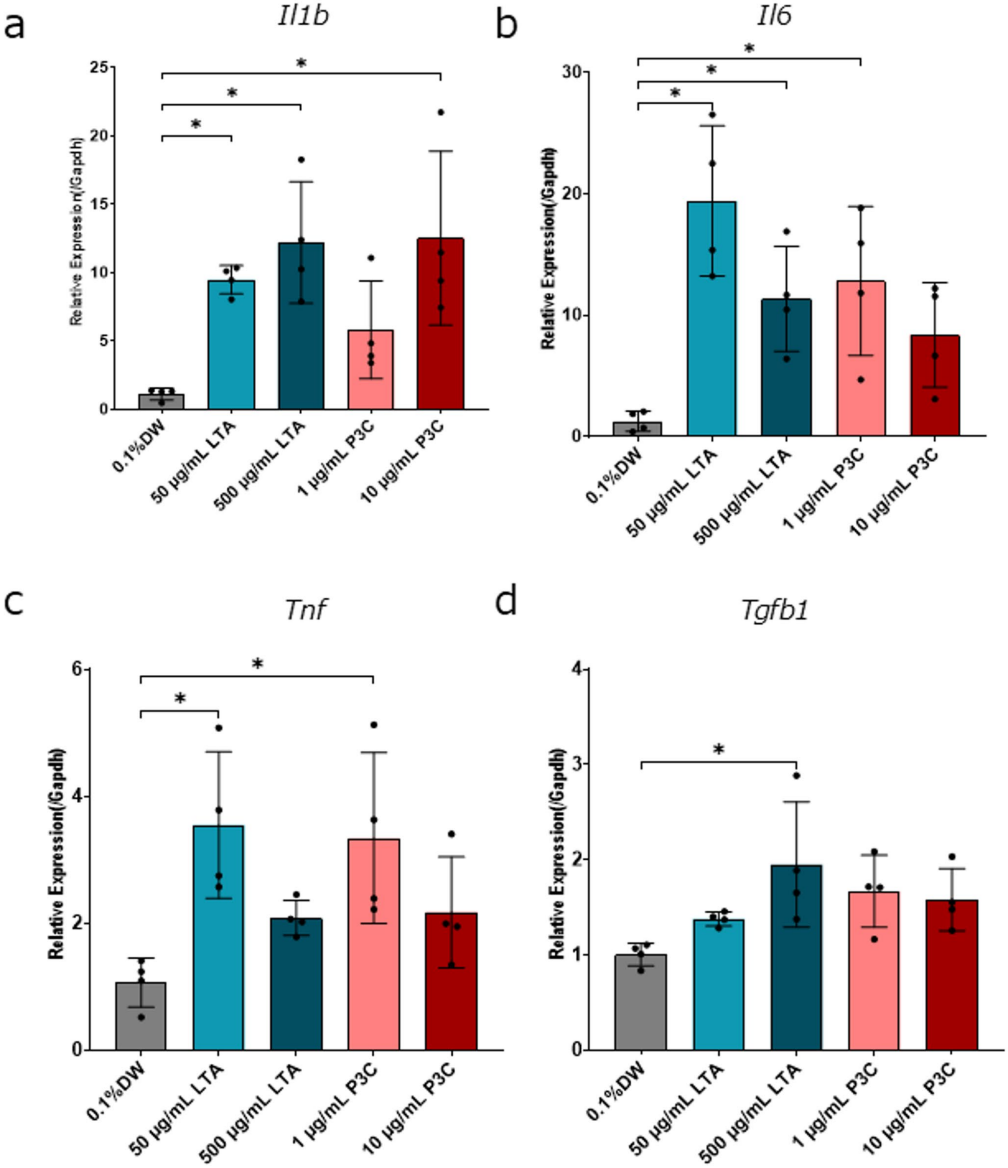
Expression of inflammatory cytokines in the cortical-derived neurons and other cells. Primary rat cortical cells were treated with TLR2 ligands and cultured for 16 days in vitro. Gene expression of the inflammatory cytokines was evaluated using qPCR. TLR2 ligands increased expression of *Il1b* (**a**), *Il6* (**b**), *Tnf* (**c**), and *Tgfb1* (**d**) ((**a**–**d**) 4 biological replicates in 1 technical replicate from 1 of 2 independent experiments). The data are presented as the mean ± SD. *n* = 4 in each experiment. *P ≤ 0.05 when relative expression levels were compared to control group, as determined by Dunnett’s test

TLR2 signaling is known to induce expression of cytokines (Dzamko et al. 2017). Based on these results, we examined the expression of cytokines in the presence of TLR2 ligands. We first cultured the cortical cells with TLR2 ligands for 16 days. Then we examined the expression of cytokines in the primary cells by qPCR. Interestingly, we found that each TLR2 ligand has different effects on the expression of cytokines. Notably, different cytokine profiles were observed between LTA and P3C (Fig. 5). Figure 5 shows that the relative expression levels of *Il1b*, *Il6*, *Tnf*, and *Tgfb1* were markedly increased by 50 µg/mL LTA. Figure 5b, c shows that there was an increase in the relative expression of *Il6* and *Tnf* by 1 µg/mL P3C. The relative expression levels of *Il1b* in the 50 µg/mL LTA group, 500 µg/mL LTA group, and in the 10 µg/mL P3C group showed significantly higher responses than that of the control group. The relative expression levels of *Il6* in the 50 µg/mL LTA group, 500 µg/mL LTA group, and in the 1 µg/mL P3C group showed significantly higher responses than that of the control group. The relative expression levels of *Tnf* in the 50 µg/mL LTA group and in the 1 µg/mL P3C group showed significantly higher responses than that of the control group. The relative expression levels of *Tgfb1* in the 500 µg/mL LTA group showed significantly higher responses than that of the control group. These data suggest that the distinct neuronal inflammatory responses were induced by several types of TLR2 ligands.

## Discussion

It has been shown that neurons and glial cells express TLR2 (Kim et al. 2015; Madar et al. 2015; Nie et al. 2018; Squillace and Salvemini 2022). Here, we show that each TLR2 ligand has effects on neuronal Ca^2 +^ dynamics and cell death. We demonstrated that LTA decreases the amplitude of the calcium spikes, and that P3C increases the number of calcium spikes. Our data show that LTA and P3C induce cell death, while LPS and ZA have no effect on Ca^2 +^ dynamics or neuronal death. To our knowledge, this is an important finding concerning the neuronal expression of TLR2 and the change in neuronal Ca2 + dynamics and cell death mediated by TLR2 ligands. Our results provide new insights into the relationship between neuroinflammation and neuronal Ca2 + dynamics.

Recent work has shown that cortical primary cells express TLR2 in humans and mice (Tang et al. 2007; Zhou et al. 2009). We showed that rat cortical neurons and non-neuronal cells express TLR2 in our rat culture system (Fig. 1). Our data indicate that TLR2 ligands can affect Ca2 + dynamics both directly and indirectly. It has been reported that TLR2 expressed by human neuronal cells is biologically functional (Zhou et al. 2009). Consistent with this report, we observe that the rat cortical cells, which are cultured in the presence of TLR2 ligands, change the Ca2 + oscillation (Fig. 3) and upregulate the gene expression of the cytokines (Fig. 5). However, a culture method that enables the detection of Ca^2 +^ spikes after completely removing astrocytes has not yet been established. Therefore, the potential influence of astrocytes on neuronal Ca^2 +^ dynamics cannot be entirely excluded. We provided evidence that the TLR2 in rat cortical cells is biologically functional. Our finding suggests that TLR2 expressed by cortical cells may react to both endogenous and exogenous antigens. It has been shown that endogenous TLR2 ligands, such as alpha-synuclein and amyloid beta can be released from damaged neurons (Zhang et al. 2023). Some pathogens that breach the blood-brain barrier can cause CNS infections (Alviz et al. 2024). A recent study has shown that the TLR3-myeloid differentiation factor 88 (MyD88) axis controls the number of spines and dendrite outgrowth in neurons (Chen et al. 2017). Our data showed that the cortical cells express TLR2 in their cell body and dendrites. The differences between how TLR2 functions in the cell body and in the dendrites remain unclear.

A previous report showed that LTA induces cerebellar glial cell death through ROS stress (Kinsner et al. 2005). Consistent with this report, we found that LTA induces cortical cell death through ROS stress. Our finding suggests that the specificity of TLR2 ligands results in the different effects on cell death. Our data suggest that simultaneous stimulation of TLR1-TLR2 and TLR2-TLR6 induces neuronal death. We clarified that immunostaining for TLR2 did not reveal marked differences in expression levels across developmental stages. Barreda et al. demonstrated that the expression of pro-caspase is significantly higher in immature neurons compared to mature ones, highlighting the developmental regulation of caspase activity during neuronal maturation (Barreda-Manso et al. 2023). When we treat cultured cells with LTA at DIV2, LDH release increases between DIV12 and DIV16. We show that LTA can induce cell death when cultured cells are treated with LTA between DIV2 and DIV9. We did not observe cell death when cultured cells were treated with LTA at DIV12 (Fig. S3). Our data suggest that LTA-induced cell death is associated with the maturation of neuronal cells. Furthermore, it has been reported that non-neuronal cells proliferate during the DIV12-16 period (Clarkson et al. 2017). In our study, early-phase stimulation with LTA induced cell death, suggesting that prolonged stimulation of TLR1/2 and TLR2/6 may lead to neuronal cell death. It has been reported that IL-6 is necessary for alpha-synuclein-mediated neuronal death (Sterling et al. 2022). We found that LTA significantly enhances the gene expression of *Il6*. MnTBAP did not fully prevent cell death. This suggests that additional, ROS-independent mechanisms may contribute, including downstream cytokine signaling through Fas-associated protein with death domain (FADD)-dependent pathways and DNA damage mediated by inflammatory factors (Liu et al. 2025).

A previous study showed that TLR2 ligands affected the concentration of Ca^2 +^ in dorsal root ganglion neurons (T.-T. Wang et al. 2020). In the present study, we demonstrated that TLR2 ligands have different effects on Ca^2 +^ frequency, Ca^2 +^ amplitude, and cell death. We found that LTA decreased the amplitude of calcium spikes, and that P3C increased the calcium spikes. We did not find that LPS and ZA affected calcium spikes (Fig. 3). TLR1-TLR2 signaling may lead to changes in Ca^2 +^ dynamics. The decrease in the amplitude of calcium spikes induced by LTA was not affected by MnTBAP (Fig. 4). When LTA was treated at DIV12, a decrease in the amplitude of calcium spikes was induced, but the release of LDH did not change. These results showed that the decrease in the amplitude of calcium spikes induced by LTA may not be due to ROS stress or neuronal death. When cultured with R-7050 from DIV2 to DIV16 to evaluate the cell death induced by LTA, the cells detached from the substrate, rendering them unsuitable for subsequent measurements. It has been reported that transforming growth factor-β (TGF-β) maintains intracellular calcium homeostasis in cortical neurons by regulating the expression of the calcium channel (Liu et al. 2018). Consistent with this report, we found that *Tgfb1* expression is enhanced (Fig. 5). Conversely, a reduction in calcium amplitude may precede cytotoxicity. Reports suggest that in pulmonary epithelial cells, suppression of calcium entry via nifedipine paradoxically elevates intracellular calcium levels, resulting in the induction of cell death (Manohar et al. 2021). Excessive neuronal activity is known to induce cell death. It has been reported that glutamate can induce excitotoxicity (Laar et al. 2015). Glutamate also triggers excitotoxicity, and the absence of Ca²⁺ in the culture medium reduces the likelihood of glutamate-induced cell death (Choi 1985). A recent study has shown that the AMPA receptor antagonist perampanel decreased the amplitude of neuronal calcium spikes and did not affect the number of spikes (Sakaguchi et al. 2023). TLR2 signaling may affect the secretion of glutamic acid and the function of AMPA receptors. Recent studies suggest that neuroinflammation regulates neuronal activity (Xanthos and Sandkühler 2014). IL-1β is associated with the hyperexcitable neuronal activity induced by focal kainate application (Vezzani et al. 1999). TNFα upregulates sensory neuron excitability via voltage-gated sodium channel (Nav) 1.7 (Tyagi et al. 2024). TNFα upregulates the excitability of the dorsal root ganglion neuron via transient receptor potential vanilloid subfamily member 1 (TRPV1) (Park et al. 2011). The induction of intercellular adhesion molecule 1 (ICAM) by IL-1β has been confirmed to be inhibited by R-7050, with its reported 50% effective concentration being approximately twice that of TNFα inhibition (Gururaja et al. 2007). The P3C-induced increase in the spikes is blocked by R-7050 (Fig. 4). Our findings serve as evidence that P3C increased the calcium spikes via IL-1β and TNFα signaling.

TLR is known to activate several distinct signaling pathways. TLR1-TLR2 dimer activates the MyD88 pathway, but TLR4 activates both the MyD88 and TRIF pathway (Kawai and Akira 2010). TLR2 ligands elicit unique cellular responses (Long et al. 2009). The cultured cortical cells in the presence of LTA increase the secretion of the cytokines (Kinsner et al. 2005). Consistent with a previous study using peripheral blood mononuclear cells (Ghosh et al. 2006), we found that P3C and LTA induced different cytokine profiles in the neuronal culture system. We observed that LTA induces the expression of *Il1b*, *Il6*, *Tnf*, and *Tgfb1* (Fig. 5). In contrast, P3C was found to induce the expression of *Il1b*, *Il6*, *Tnf*, but not *Tgfb1*. Our data suggest that TLR2 dimer-specificity could induce the specific expression of cytokines by cortical cells. It remains unclear what type of cells express these cytokines. These cytokines were investigated for their potential involvement in cell death and neural activity. In dorsal root ganglion (DRG) neurons, inhibition of IL-1β signaling has been reported to attenuate caspase 3–induced neuronal death, indicating a pro-degenerative role of IL-1β signaling in certain neuronal contexts (Wang et al. 2005). In hippocampal neurons, IL-1β stimulation increases NR2A/NR2B (GluN2A/2B) expression, augments N-methyl-D-aspartate (NMDA) receptor–mediated neuronal activity, and upregulates IL-6 expression, suggesting that neuronally relevant IL-1β signaling can modulate synaptic responsiveness and secondarily influence cytokine networks that may affect homeostatic set points (Viviani et al. 2003). Under conditions of NMDA-induced excitotoxic stress, IL-6 has been shown in cerebellar granule neurons to reduce neuronal death, consistent with a context-dependent neuroprotective action (Ma et al. 2015). Simultaneously, IL-6 can potentiate the transient increase in intracellular calcium following NMDA application, implying that IL-6 may reshape Ca^2 +^ dynamics and downstream signaling without necessarily exacerbating cell death, depending on timing, dose, and receptor engagement (Qiu et al. 1995). DRG neurons express TNF receptors that can synergize with AMPA receptor signaling to amplify Ca^2 +^ influx (Li et al. 2023). During low-potassium–induced apoptosis of cerebellar granule neurons, TGF-β has been reported to exacerbate cell death (de Luca et al. 1996), suggesting that TGF-β is not uniformly neuroprotective and may promote pro-apoptotic pathways under specific conditions (Vivien and Ali 2006). We have incorporated this nuance to emphasize that neuronally relevant TGF-β signaling can be bidirectional with respect to survival, contingent on developmental stage, injury state, and signaling context (Meyers and Kessler 2017).

In conclusion, we have demonstrated that the TLR2 ligands have effects on Ca^2 +^ dynamics and cell death. LTA may lead to cell death through ROS signaling. P3C is likely to contribute to the increase in Ca^2 +^ dynamics via IL-1β and TNFα signaling. It is possible that the cytokine profiles induced by TLR2 ligands result in different reactions in cultured cortical cells. These findings will advance our understanding of the complex role of TLR2 in CNS, and it remains an important task to determine how cytokines alter Ca^2 +^ dynamics. Our findings demonstrate that TLR2 ligands influence intracellular calcium dynamics, which are closely associated with neuronal signaling processes. However, since calcium imaging does not directly measure action potentials or synaptic transmission, future studies incorporating electrophysiological recordings will be essential to determine whether TLR2 activation directly modulates neuronal activity.

## Supplementary Information

Below is the link to the electronic supplementary material.

Overview of the experimental workflow. The conditions for compound addition in the experiments conducted for each figure are described. Supplementary Material 1

Confirmation of non-neuronal markers in this culture system. Primary rat cortical cells were cultured for 16 days. (a) Representative images showing the signal from Hoechst 33342-stained nuclei in blue, beta III tubulin immunoreactivity in green, and GFAP, Iba1, Olig2 immunoreactivity in red in the cultured cortical cells, and merged image (bottom panel). 4 biological replicates in 1 technical replicate from 1 independent experiment. Scale bar: 100 µm. Cells were analyzed in 4 fields of view. Supplementary Material 2

The time course of LTA-induced cell death. Release of LDH was measured photometrically as a surrogate for cell death at DIV16. (a) The cells were treated with 50 µg/mL LTA at DIV2, 5, 7, 9, and LDH measurement conducted at DIV16. (b) The cells were treated with 50 µg/mL LTA at DIV2, 12 and LDH measurement conducted at DIV16. (a) The plots indicate the mean and the data of each well. 2 biological replicates in 1 technical replicate in the experiment. Statistical testing was not performed. (b) The data are presented as the mean ± SD. 4 biological replicates in 1 technical replicate in the experiment. *P < 0.05 vs. DIV2–16 control group as determined by Tukey’s test. Supplementary Material 3

The waveforms of calcium oscillations affected by TLR ligands. The actual waveforms of calcium oscillations are presented, with one well shown per group. (a) The cells were treated with LTA at DIV2. (b) The cells were treated with P3C or ZA at DIV12. Supplementary Material 4

## Data Availability

The datasets generated during the current study are not publicly available but are available from the corresponding author on reasonable request.

## Abbreviations

BSA: Bovine serum albumin
CNS: Central nervous system
DIV: Days in vitro
FADD: Fas-associated protein with death domain
GFAP: Glial fibrillary acidic protein
HBSS: Hanks’ balanced salts solution
ICAM: Intercellular adhesion molecule 1
IL: Interleukin
JAK: Janus kinase
LDH: Lactate dehydrogenase
LPS: Lipopolysaccharide
LTA: Lipoteichoic acid
MyD88: Myeloid differentiation factor 88
Nav: Voltage-gated sodium channel
NMDA: N-methyl-D-aspartate
P3C: Pam3CSK4
PAMPs/DAMPs: Pathogen/Damage-associated molecular patterns
PBS: Phosphate-buffered saline
PFA: Paraformaldehyde
ROS: Reactive oxygen species
PRR: Pattern recognition receptor
TRPV1: Transient receptor potential vanilloid subfamily member 1
TNFα: Tumor necrosis factor alpha
TUBB3: Class III beta-tubulin
TLR: Toll-like receptor
ZA: Zymosan A
