# Supplementary figures and images for: Diverse Effects of Various Toll-Like Receptor 2 Ligands on Neuronal Activity and Cell Death
Source: Cell Mol Neurobiol. 2025 Nov 26;46:4. doi: 10.1007/s10571-025-01632-3 (PMC12775245; doi:10.1007/s10571-025-01632-3)

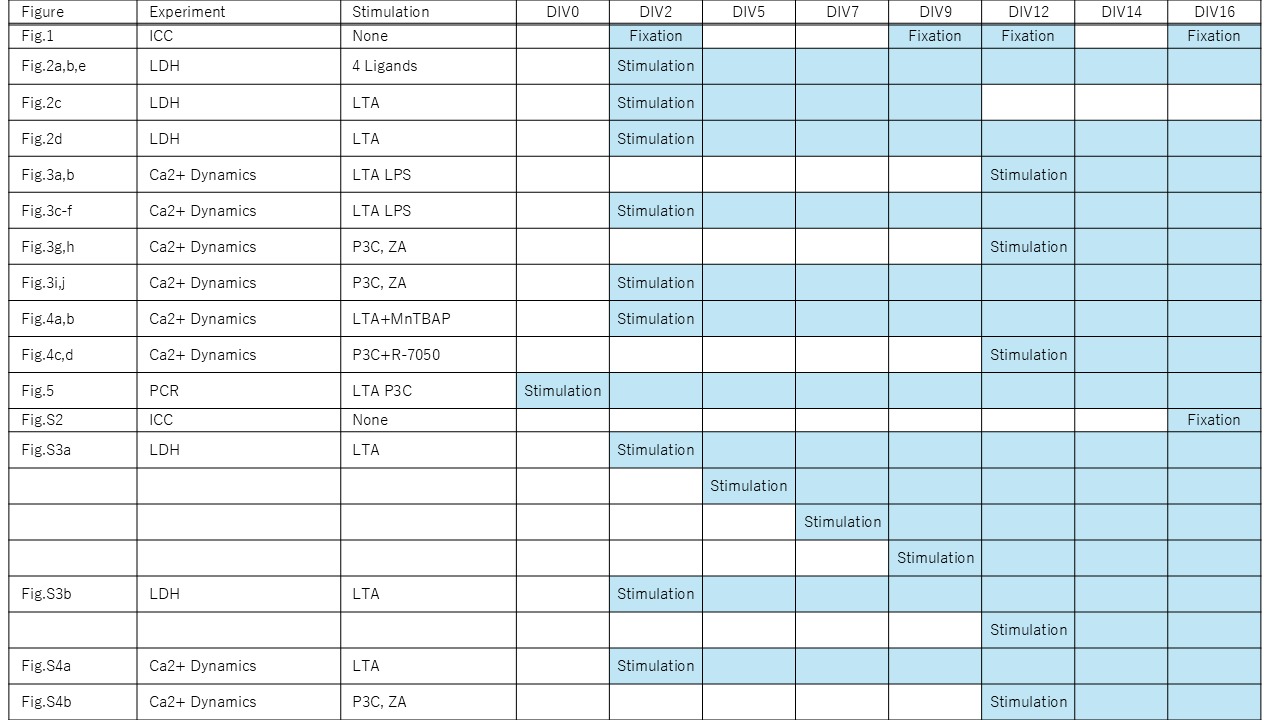

Supplement: Supplementary file 1 — Overview of the experimental workflow. The conditions for compound addition in the experiments conducted for each figure are described. Supplementary Material 1 [file 10571_2025_1632_MOESM1_ESM.tif]

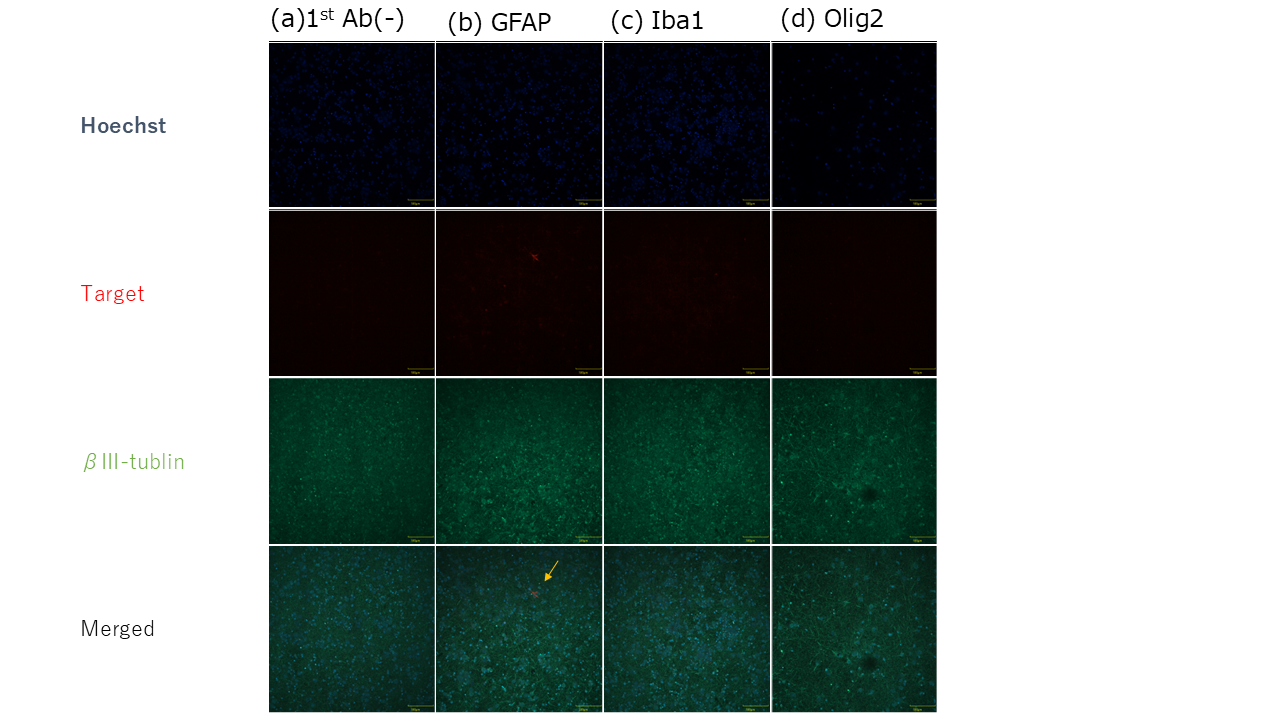

Supplement: Supplementary file 2 — Confirmation of non-neuronal markers in this culture system. Primary rat cortical cells were cultured for 16 days. (a) Representative images showing the signal from Hoechst 33342-stained nuclei in blue, beta III tubulin immunoreactivity in green, and GFAP, Iba1, Olig2 immunoreactivity in red in the cultured cortical cells, and merged image (bottom panel). 4 biological replicates in 1 technical replicate from 1 independent experiment. Scale bar: 100 µm. Cells were analyzed in 4 fields of view. Supplementary Material 2 [file 10571_2025_1632_MOESM2_ESM.tif]

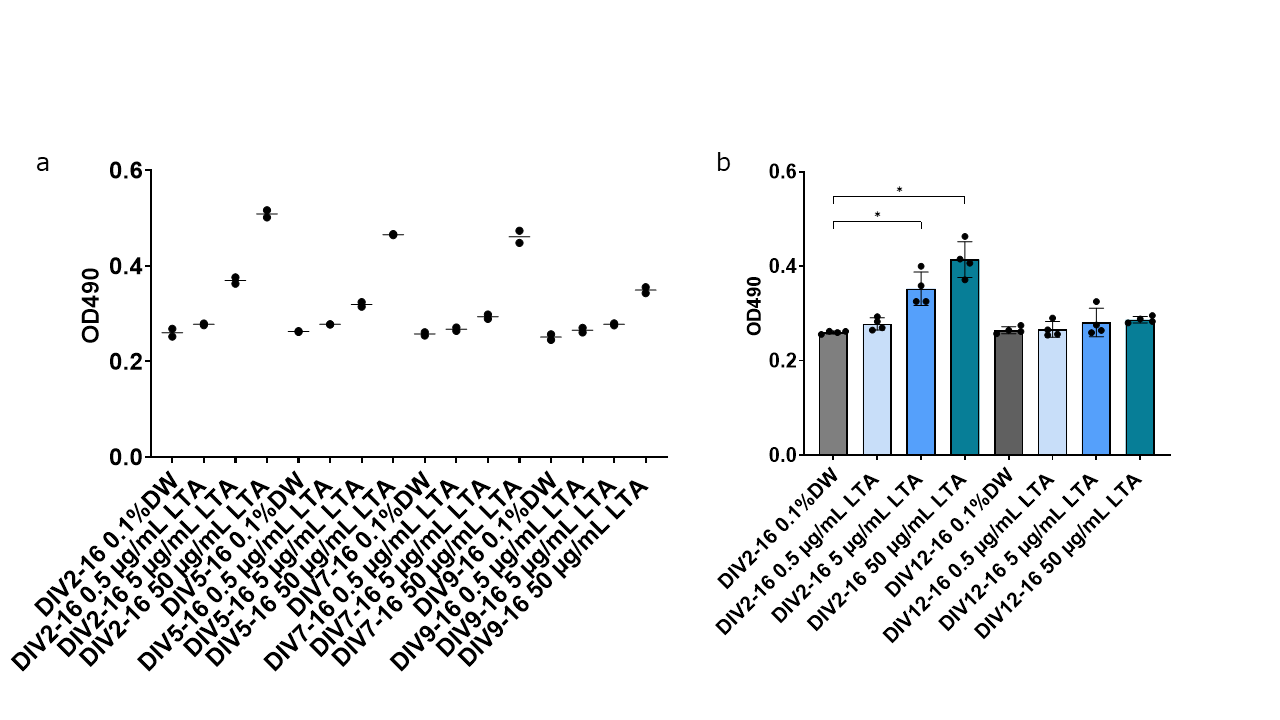

Supplement: Supplementary file 3 — The time course of LTA-induced cell death. Release of LDH was measured photometrically as a surrogate for cell death at DIV16. (a) The cells were treated with 50 µg/mL LTA at DIV2, 5, 7, 9, and LDH measurement conducted at DIV16. (b) The cells were treated with 50 µg/mL LTA at DIV2, 12 and LDH measurement conducted at DIV16. (a) The plots indicate the mean and the data of each well. 2 biological replicates in 1 technical replicate in the experiment. Statistical testing was not performed. (b) The data are presented as the mean ± SD. 4 biological replicates in 1 technical replicate in the experiment. *P < 0.05 vs. DIV2–16 control group as determined by Tukey’s test. Supplementary Material 3 [file 10571_2025_1632_MOESM3_ESM.tif]

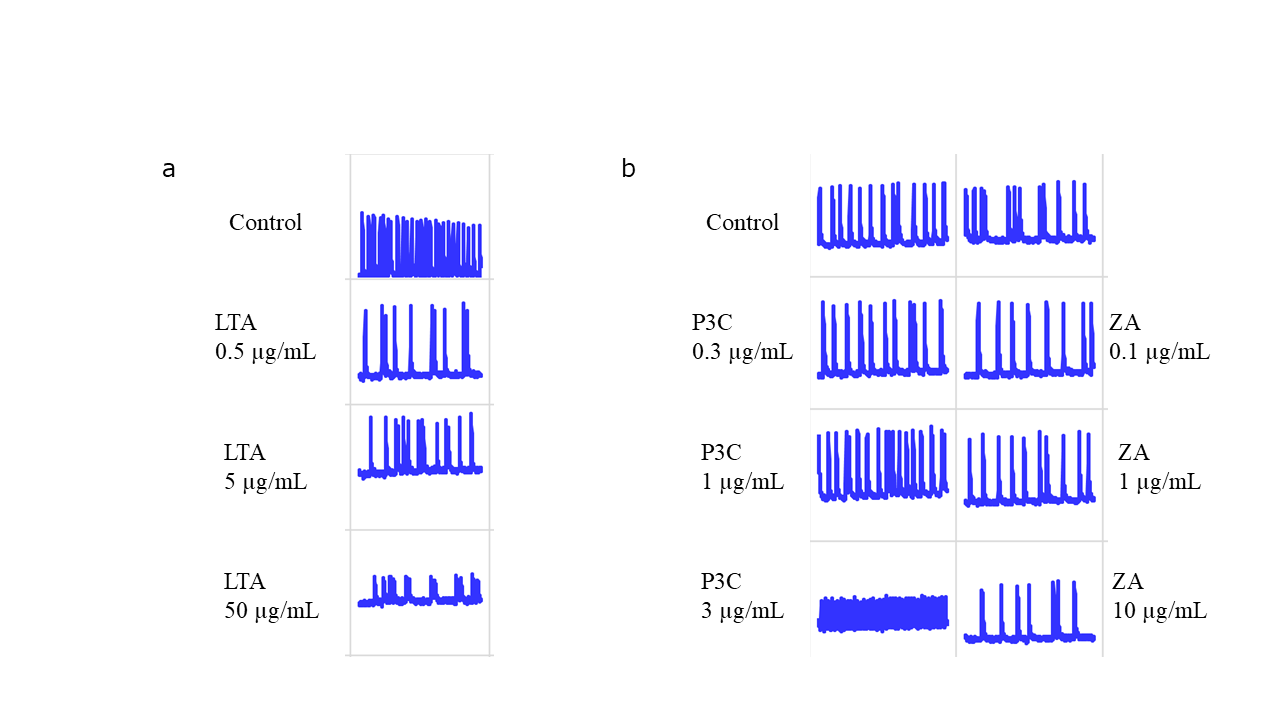

Supplement: Supplementary file 4 — The waveforms of calcium oscillations affected by TLR ligands. The actual waveforms of calcium oscillations are presented, with one well shown per group. (a) The cells were treated with LTA at DIV2. (b) The cells were treated with P3C or ZA at DIV12. Supplementary Material 4 [file 10571_2025_1632_MOESM4_ESM.tif]
